# Müllerian anomalies in girls with congenital solitary kidney

**DOI:** 10.1007/s00467-023-06266-5

**Published:** 2024-01-10

**Authors:** Laura Walawender, Natasha Santhanam, Benjamin Davies, Y. Frances Fei, Daryl McLeod, Brian Becknell

**Affiliations:** 1 Kidney and Urinary Tract Center, Abigail Wexner Research Institute at Nationwide Children’s Hospital, 700 Children’s Drive, Columbus, OH 43205 USA; 2 Division of Nephrology and Hypertension, Nationwide Children’s Hospital, Columbus, OH USA; 3 Section of Pediatric and Adolescent Gynecology, Nationwide Children’s Hospital, 700 Children’s Drive, Columbus, OH 43205 USA; 4 Department of Urology, Nationwide Children’s Hospital, Columbus, OH USA

**Keywords:** Kidney, Uterus, Vagina, Congenital, Malformations, CAKUT, Solitary kidney, Unilateral renal agenesis, Multicystic dysplastic kidney, Müllerian anomalies, Complications

## Abstract

**Background:**

The prevalence of Müllerian anomalies (MA) among patients with congenital solitary functioning kidney (SFK) is not well defined. A delay in diagnosis of obstructive MA can increase the risk of poor clinical outcomes. This study describes the prevalence of MA in patients with congenital SFK.

**Methods:**

A retrospective review was performed of patients within the Nationwide Children’s Hospital system with ICD9 or ICD10 diagnostic codes for congenital SFK defined as either unilateral renal agenesis (URA) or multicystic dysplastic kidney (MCDK) and confirmed by chart review. Patients with complex urogenital pathology were excluded. Renal anomaly, MA, reason for and type of pelvic evaluation, and age of diagnosis of anomalies were evaluated.

**Results:**

Congenital SFK occurred in 431 girls due to URA (209) or MCDK (222). Pelvic evaluation, most commonly by ultrasound for evaluation of abdominal pain or dysmenorrhea, occurred in 115 patients leading to MA diagnosis in 60 instances. Among 221 patients ages 10 years and older, 104 underwent pelvic evaluation and 52 were diagnosed with an MA of which 20 were obstructive. Isolated uterine or combined uterine and vaginal anomalies were the most common MA. MA were five-fold more common in patients with URA compared to MCDK. In 75% of patients, the SFK was diagnosed prior to the MA.

**Conclusions:**

The prevalence of MA in patients with congenital SFK was 24% among those age 10 years or older, and 38% were obstructive. This justifies routine screening pelvic ultrasound in girls with congenital SFK to improve early diagnosis.

**Graphical abstract:**

**Figure:**
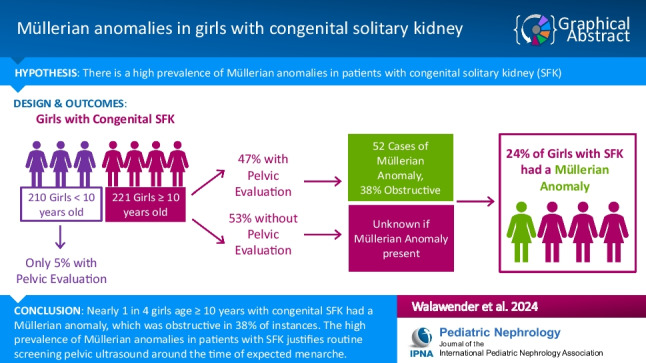
A higher resolution version of the Graphical abstract is available as Supplementary information

**Supplementary Information:**

The online version contains supplementary material available at 10.1007/s00467-023-06266-5.

## Introduction

It is well known that Müllerian and urinary tract development are inter-related. Accordingly, there is an increased incidence of renal anomalies — particularly unilateral renal agenesis (URA) — in patients with a known Müllerian anomaly (MA) [1–3]. Conversely, the risk of MA among patients with a known congenital solitary functioning kidney (SFK) — either URA or multicystic dysplastic kidney (MCDK) — is not well established, though frequencies of 32–67% have been reported [4, 5]. A 2021 study of females with a known renal anomaly demonstrated a 29% risk of co-existing MA. In this study, only 21% of patients were screened for an MA at the time of their renal anomaly diagnosis [5]. In a 2022 study of post-menarchal females, 38% of patients with a known renal anomaly who underwent pelvic evaluation were also found to have an MA [4]. The majority of these patients had an SFK due to URA or MCDK, including nearly half of whom had an obstructive MA requiring urgent surgical management [4]. Most MA are not diagnosed until adulthood when infertility or persistent dysmenorrhea leads to evaluation, though obstructive lesions are often diagnosed around the time of menarche due to acute symptoms, such as severe abdominal pain [2]. A delay in diagnosis of obstructive MA can lead to poor clinical outcomes including pelvic inflammatory disease, tubo-ovarian abscesses, endometriosis, and infertility [5].

Due to routine prenatal ultrasounds, it is common to identify an SFK prior to birth. Approximately 1 in 2200 births are affected by URA, whereas unilateral MCDK occurs in 1 in 4200 births [6]. Postnatal ultrasound can confirm the diagnosis, and subsequent evaluation and follow-up vary greatly based on local practice patterns [7]. New guidelines for management of patients with SFK recommend screening female patients for MA, but the ideal age of screening is not yet known or agreed upon [6, 8]. Given that an SFK is typically identified well before menarche, routine screening for MA prior to acute presentation should be feasible. Ultrasound is the preferred method for initial screening for MA, but MRI is ultimately the gold standard for anatomical evaluation [9, 10]. Evaluation for MA by advanced imaging typically needs to occur after thelarche when sufficient hormonal stimulation of the uterus has occurred to allow visualization of abnormalities and obstructive lesions. Alternatively, it has been suggested that maternal hormonal stimulation of the genitourinary tract may be sufficient for diagnosis of MA as a neonate [6]; however, this is not universally accepted. Therefore, diligence is required by the provider and family to obtain pelvic imaging many years after the renal anomaly diagnosis.

The objective of this study was to understand the prevalence of MA in patients with congenital SFK. Additional aims were to describe the types of MA, the frequency of obstructive anomalies, and the relative timing of diagnosis of these anomalies.

## Methods

This is a retrospective cohort study of female patients with a known diagnosis of SFK seen at Nationwide Children’s Hospital between 1/1/2008 and 12/31/2022. Nationwide Children’s Hospital is a tertiary referral center with dedicated pediatric nephrologists, urologists, and gynecologists. After IRB approval (STUDY00002901) patients with SFK were identified using ICD9 (753, 753.19, 753.15) and ICD10 (Q60.0, Q61.4, Q60.3) codes. Patients with complex urogenital pathology, such as, anorectal malformation, cloaca, urogenital sinus, or bladder exstrophy, were excluded. Basic patient data including race, age of last visit to a health care provider, renal diagnosis, MA diagnosis, age of diagnoses, reason for pelvic evaluation, type of pelvic evaluation, and treatment were collected. Patients were considered to have a pelvic evaluation if there was imaging or surgical evaluation dedicated to the pelvic structures with clear commentary on the report that the structures were visualized.

Renal anomaly diagnosis was confirmed by chart review. SFK diagnosis was categorized as either URA or MCDK. Patients with an acquired SFK due to nephrectomy were excluded. Patients with history of nephrectomy of an MCDK were included.

MA diagnosis was recorded and classified as either a uterine anomaly (hypoplasia/agenesis, unicornuate, didelphys, bicornuate, septate, arcuate), a vaginal anomaly (oblique septum (OHVIRA), longitudinal septum, transverse septum, partial vaginal agenesis/distal vaginal atresia), or both uterine and vaginal anomalies present. This classification is in line with the 2021 Müllerian anomalies classification system developed by the American Society for Reproductive Medicine [11]. Treatment of obstructive MA was categorized in three groups: “urgent surgical management” if performed within 2 weeks of diagnosis, “hormonal suppression” if treated initially with menstrual suppression even though they may have undergone eventual surgical management, and “delayed surgical management” if patients were relatively asymptomatic and did not require urgent surgery or immediate hormonal suppression.

The patients with SFK were subdivided after data collection into two groups — those age 0 to 9 years and 364 days old at the time of last visit, and those age 10 years or older at the time of last visit. Age 10 was chosen given that this is the earliest typical onset of menarche [12].

## Results

**Fig. 1:**
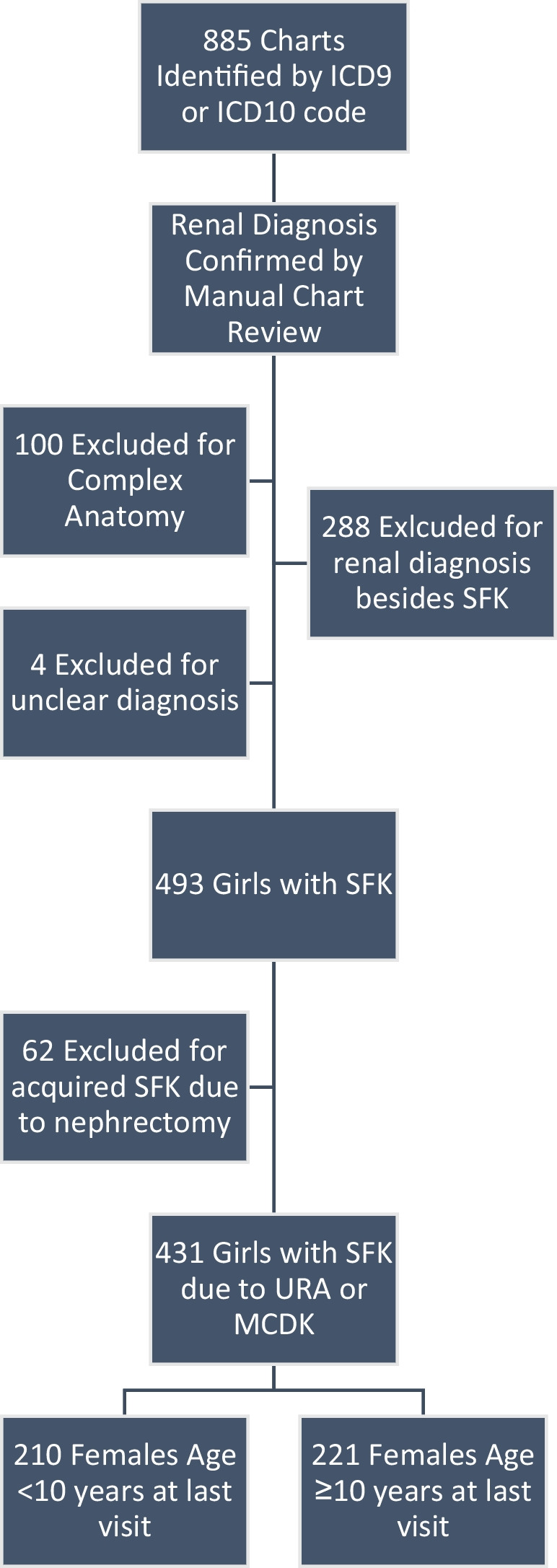
Flow diagram to determine patient eligibility

We identified 885 subjects by diagnostic codes for SFK. Two hundred eighty-eight were excluded for renal diagnoses other than SFK; 100 were excluded due to complex urogenital anatomy; an additional 62 were excluded due to acquired SFK secondary to nephrectomy; and 4 were excluded due to diagnostic uncertainty (Fig. 1). This left 431 girls with congenital SFK due to URA (209) or MCDK (222) (Table 1).

**Table 1:** Characteristics of girls with congenital SFK

Characteristic	All patients (431)	Age < 10 years (210)	Age ≥ 10 years (221)
Race			
White or Caucasian	283 (65%)	127 (60%)	156 (70%)
Black or African American	55 (13%)	26 (12%)	29 (13%)
Asian	20 (5%)	14 (7%)	6 (3%)
Mixed race	35 (8%)	21 (10%)	14 (6%)
Latino/Hispanic	20 (5%)	12 (6%)	8 (4%)
Other^	18 (4%)	10 (5%)	8 (4%)
Congenital SFK diagnosis			
URA	209 (48%)	77 (37%)	132 (60%)
MCDK	222 (52%)	133 (63%)	89 (40%)
Age of SFK diagnosis*	0, 0–0	0, 0–0	0, 0–6
Age of last clinic visit*	10, 4–17	4, 2–7	16, 13–20

^African, Native Hawaiian and other Pacific Islander, other, unknown

^*^Median age in years, interquartile range (IQR)

Among these subjects, 115 (27%) underwent pelvic evaluation (Table 2), yielding an MA diagnosis in 60 instances (14% of the entire cohort) at a median age of 14.5 years (IQR 12–16). Most commonly, the MA was identified as either a combined abnormality of both the uterus and vagina or an isolated uterine anomaly. Isolated vaginal anomalies were rare (Table 3). The SFK was diagnosed either prenatally or within the first year of life for 72% of patients. Among patients with an MA, the congenital SFK was diagnosed prior to the MA in 76% of instances, and the underlying renal anomaly was URA in 83% of cases.

**Table 2:** Details of pelvic evaluation*

Characteristic	All (115)	URA (84)	MCDK (31)	Age < 10 (11)		Age < 10 with URA (7)	Age < 10 with MCDK (4)	Age ≥ 10 (104)	Age ≥ 10 with URA (77)	Age ≥ 10 with MCDK (27)
Pelvic evaluation										
Uterine MA	29 (25%)	22 (26%)	7 (22%)	6 (55%)	4 (57%)		2 (50%)	23 (22%)	18 (23%)	5 (19%)
Vaginal MA	2 (2%)	2 (2%)		1 (9%)	1 (14%)			1 (1%)	1 (1%)	
Combined uterine/vaginal MA	29 (25%)	26 (31%)	3 (10%)	1 (9%)	1 (14%)			28 (27%)	25 (33%)	3 (11%)
No MA	55 (48%)	34 (41%)	21 (68%)	3 (27%)	1 (14%)		2 (50%)	52 (50%)	33 (43%)	19 (70%)
Type of pelvic evaluation										
Ultrasound	84 (73%)	60 (71%)	24 (77%)	9 (82%)	6 (86%)		3 (75%)	75 (72%)	54 (70%)	21 (78%)
CT	37 (32%)	31 (37%)	6 (19%)					37 (36%)	31 (40%)	6 (22%)
MRI	40 (35%)	33 (39%)	7 (23%)	4 (36%)	2 (29%)		2 (50%)	36 (35%)	31 (40%)	5 (19%)
Surgery	4 (3%)	3 (4%)	1 (3%)	1 (9%)	1 (14%)			3 (3%)	2 (3%)	1 (4%)
Unknown	4 (3%)	4 (5%)		1 (9%)	1 (14%)			3 (3%)	3 (4%)	
Indication for pelvic evaluation										
Routine screening	1 (1%)		1 (3%)					1 (1%)		1 (4%)
Abdominal pain/dysmenorrhea	40 (35%)	33 (39%)	7 (2%)					40 (38%)	33 (43%)	7 (26%)
Abnormal uterine bleeding	12 (10%)	12 (14%)						12 (12%)	12 (16%)	
Finding on renal ultrasound	11 (10%)	4 (5%)	7 (2%)	4 (36%)	1 (14%)		3 (75%)	7 (7%)	3 (4%)	4 (15%)
Finding during other work up	15 (13%)	13 (15%)	2 (6%)	4 (36%)	4 (57%)			11 (11%)	9 (12%)	2 (7%)
Pregnancy	4 (3%)	3 (4%)	1 (3%)					4 (4%)	3 (4%)	1 (4%)
Primary amenorrhea	8 (7%)	6 (7%)	2 (6%)					8 (8%)	6 (8%)	2 (7%)
Finding during surgery	2 (2%)	2 (2%)						2 (2%)	2 (3%)	
Physical exam	8 (7%)	7 (8%)	1 (3%)	2 (18%)	2 (29%)			6 (6%)	5 (6%)	1 (4%)
Infertility evaluation	1 (1%)	1 (1%)						1 (1%)	1 (1%)	
Other	21 (18%)	10 (12%)	11 (35%)	1 (9%)			1 (25%)	20 (19%)	10 (13%)	10 (37%)

^*^Some patients underwent > 1 form of pelvic evaluation and/or had > 1 indication for pelvic evaluation

**Table 3:** Characteristics of MA

Characteristic	All (60)	URA (50)	MCDK (10)	Age < 10 (8)	Age < 10 with URA (6)	Age < 10 with MCDK (2)	Age ≥ 10 (52)	Age ≥ 10 with URA (44)	Age ≥ 10 with MCDK (8)
First diagnosed anomaly									
SFK	42 (70%)	32 (64%)	10/10 (100%)	6 (75%)	4 (66%)	2 (100%)	36 (69%)	28 (64%)	8 (100%)
MA	5 (8%)	5 (10%)					5 (10%)	5 (11%)	
Simultaneously	8 (13%)	8 (16%)		1 (12.5%)	1 (17%)		7 (13%)	7 (16%)	
Unknown	5 (8%)	5 (10%)		1 (12.5%)	1 (17%)		4 (8%)	4 (9%)	
Uterine anomaly									
Hypoplasia/agenesis	9 (15%)	8 (16%)	1 (10%)				9 (17%)	8 (18%)	1 (13%)
Unicornuate	9 (15%)	9 (18%)		1 (13%)	1 (17%)		8 (15%)	8 (18%)	
Didelphys	26 (43%)	23 (46%)	3 (30%)	2 (25%)	2 (33%)		24 (46%)	21 (48%)	3 (38%)
Bicornate	9 (15%)	5 (10%)	4 (40%)	4 (50%)	2 (33%)	2 (100%)	5 (10%)	3 (7%)	2 (25%)
Septate	3 (5%)	2 (4%)	1 (10%)				3 (6%)	2 (5%)	1 (13%)
Arcuate	2 (3%)	1 (2%)	1 (10%)				2 (4%)	1 (2%)	1 (13%)
Vaginal anomaly									
Oblique septum	18 (30%)	16 (32%)	2 (20%)	2 (25%)	2 (33%)		16 (31%)	14 (32%)	2 (25%)
Longitudinal septum	3 (5%)	3 (6%)					3 (6%)	3 (7%)	
Transverse septum	1 (2%)	1 (2%)					1 (2%)	1 (2%)	
Partial agenesis/distal atresia	9 (15%)	8 (16%)	1 (10%)				9 (17%)	8 (16%)	1 (13%)
Müllerian obstruction	22 (37%)	20 (40%)	2 (20%)	2 (25%)	2 (33%)		20 (38%)	18 (41%)	2 (25%)
Urgent surgery	13 (59%)	11 (55%)	2 (100%)				13 (65%)	11 (61%)	2 (100%)
Hormonal suppression	5 (9%)	5 (25%)					5 (25%)	5 (28%)	
Delayed surgery	4 (18%)	4 (20%)		2 (100%)	2 (100%)		2 (10%)	2 (11%)	
Age of MA diagnosis*	14.5, 12–16	14.5, 13–16	14, 10–19	0, 0–1	0, 0–0.75	0.5, 0.25–0.75	15, 13–16	15, 13–16	16, 13–20

^*^Median age in years, IQR

A subanalysis was performed of girls who were more likely to have experienced onset of puberty, using 10 years of age as a cutoff [12], under the rationale that girls of menarchal age would more likely experience symptoms and undergo pelvic evaluation, acknowledging that the timing of puberty onset was not available from chart review. Consistent with this reasoning, among the 210 patients ages less than 10 years at the time of last clinic visit, only 11 (5%) patients underwent pelvic evaluation, leading to an MA diagnosis in eight cases. Conversely, among the 221 girls ages 10 years or older at the time of last clinic visit, 104 (47%) underwent pelvic evaluation, yielding an MA diagnosis in 52 cases. The most common reason for pelvic evaluation was abdominal pain or dysmenorrhea, and ultrasound was the most commonly utilized imaging modality (Table 2). Thirty-eight percent of patients ages 10 years or older with MA experienced menstrual obstruction, and 65% of these cases required urgent surgical intervention (Table 3). Of patients age 10 years and older, the median age of SFK diagnosis was 0 years (IQR 0–0) and the median age of MA diagnosis was 15 years (IQR 13–16). Seventy-five percent of girls ages 10 years or older were diagnosed with congenital SFK prior to MA diagnosis.

## Discussion

While it is broadly appreciated that MA are associated with increased frequency of congenital SFK, few studies have addressed the prevalence of MA among patients with congenital renal anomalies including SFK. The goal of this study was to determine the prevalence of MA among girls with a known diagnosis of congenital SFK. As far as we are aware, this is the largest study that focuses specifically on patients with SFK. When evaluating our entire cohort, there was a prevalence of 14% of MA in girls with congenital SFK. Among patients ≥ 10 years old, the prevalence of MA was 24%. In this older cohort, the MA was obstructive in 38% of instances, and 65% of obstructive MA required urgent surgical intervention.

Our cohort included a large portion of patients who were pre-pubertal (ages less than 10 years) at the time of evaluation, and only 5% of this cohort underwent pelvic evaluation. We therefore felt it more clinically relevant to focus on the older cohort (ages 10 years and older) who are expected to have experienced onset of puberty. Within this cohort, almost half underwent pelvic evaluation, one-third for abdominal pain or dysmenorrhea; only one of the 221 patients underwent pelvic imaging for the purpose of screening. Even if we assume that all patients who did not undergo pelvic evaluation were negative for MA, the minimum prevalence of MA among girls age ≥ 10 years would still be 24%, which is a significant proportion of girls with SFK. Depending on the MA, some diagnoses can be asymptomatic until conception is desired, meaning that without routine screening these diagnoses would not be made until closer to adulthood. Therefore, it is likely that more patients within this group would be confirmed to have an MA if pelvic imaging were obtained. Accordingly, the true prevalence is likely higher than 24%. This is overall consistent with recent studies which have shown a prevalence ranging from 32 to 67% [4, 5].

Even more importantly, the prevalence of obstructive lesions among girls ages 10 years and older with MA was 38%, and nearly two-thirds of cases required urgent surgical intervention. Obstructive MA can lead to significant effects on future fertility and reproductive health such as ascending infection causing pelvic inflammatory disease or tubo-ovarian abscess, endometriosis, tubal injury, pelvic adhesions, or need for sterilizing surgery [1, 13]. It is therefore critical to avoid delays in diagnosis of obstructive MA. Patients are often diagnosed with obstructive lesions during adolescence due to acute symptomatology, though diagnosis can also be delayed until adulthood due to their non-specific presentation [1, 14].

Given the significant morbidity associated with delayed diagnosis of MA, our study emphasizes the need for routine pelvic imaging to screen for MA among patients with congenital SFK, as advocated by recent publications [1, 4–6, 8]. In our cohort, most patients were diagnosed with SFK either prenatally or within the first year of life and three-fourths of patients had their SFK diagnosed before their MA. This suggests an opportunity to implement screening to preemptively detect MA and avoid a delay in diagnosis which can have detrimental effects on future reproductive health [13].

A recent guideline for management of patients with congenital SFK suggests that pelvic imaging to screen for MA could be obtained at the time of birth due to maternal estrogen stimulation [6]. Maternal estrogen exposure can lead to some enlargement of the uterus, as well as prepubertal vaginal bleeding, which usually self-resolves within the first 1–2 weeks after birth [15]. Therefore, it is possible to detect an MA if imaging is performed within the first 2 weeks of life, though the sensitivity of this screening is unknown. Screening for MA in the prepubertal period after the initial 1–2 weeks of life becomes less reliable as the uterus reverts to a prepubertal size and shape [10].

Alternatively, in view of these limitations, screening for MA can occur via transabdominal pelvic ultrasound 2–3 years after thelarche or around the age of expected menarche, which occurs at 12–12.5 years [12]. Since pelvic imaging in early adolescence would occur many years after the SFK diagnosis was made in infancy, this raises the question of whether responsibility for screening falls to the child’s primary care provider or pediatric nephrologist. The frequency of office visits that patients with congenital SFK have with a pediatric nephrologist varies substantially depending on patient-specific factors and institutional practices [7], and in some cases medical care may be exclusively provided by primary care providers. It is therefore critical that both generalists and specialists caring for this population are aware of the high prevalence of coexisting congenital SFK and MA and employ adequate screening at the appropriate time in development.

In our study, the prevalence of MA was five-fold higher in patients with URA compared to MCDK. While URA and MCDK are the two etiologies of congenital SFK, the underlying embryonic abnormality is fundamentally different [16]. URA results from an insult early in the development process leading to complete aplasia whereas MCDK represents a form of dysplasia generally thought to occur secondary to disruptions in nephrogenesis [17]. It has previously been shown that URA when compared to MCDK is associated with additional abnormalities in the SFK, extra-renal anomalies, an underlying genetic syndrome, and progression of CKD [18]. Thus, the higher prevalence of MA in patients with URA is not surprising. However, both populations have a high prevalence of MA and therefore all patients with SFK should be screened for MA, in accordance with recent guidelines [6, 8]. The prevalence of MA in girls with other congenital anomalies of the kidney and urinary tract has not been well described, and more research is needed to determine if routine pelvic screening should be recommended in such instances.

This investigation was limited by the inherent biases of a retrospective single-center study. We focused on girls with SFK due to either URA or MCDK, and it is possible that some of the patients classified as URA previously had MCDK that involuted prior to the time of SFK diagnosis. The prevalence of MA may have been over-estimated, because those presenting with symptoms are more likely to undergo imaging, representing a selection bias, which likely explains why 50% of patients ages 10 and older who had pelvic imaging were found to have an MA. Nonetheless, the minimum prevalence of MA among girls with SFK age 10 years or older was 24% when considering that 52 of the 221 girls were found to have an MA, and this likely underestimates the actual prevalence that would have been detected through universal screening. In conclusion, our findings support the need for MA screening by pelvic ultrasound in patients with congenital SFK — ideally 2 to 3 years after the onset of thelarche around the age of expected menarche. Further multicenter studies are required to substantiate our findings and to follow the outcomes of universal screening programs once they are instituted.

### Supplementary Information

Below is the link to the electronic supplementary material.

Graphical Abstract (PPTX 63 KB)

## Data Availability

The datasets generated during this study are available from the corresponding authors upon request.
