# Supplementary material for: Müllerian anomalies in girls with congenital solitary kidney
Source: Pediatr Nephrol. 2024 Jan 10;39(6):1783–9. doi: 10.1007/s00467-023-06266-5 (PMC11026257; doi:10.1007/s00467-023-06266-5)
Supplement: Supplementary file 1 — Graphical Abstract (PPTX 63 KB) [file 467_2023_6266_MOESM1_ESM.pptx]

## Slide 1
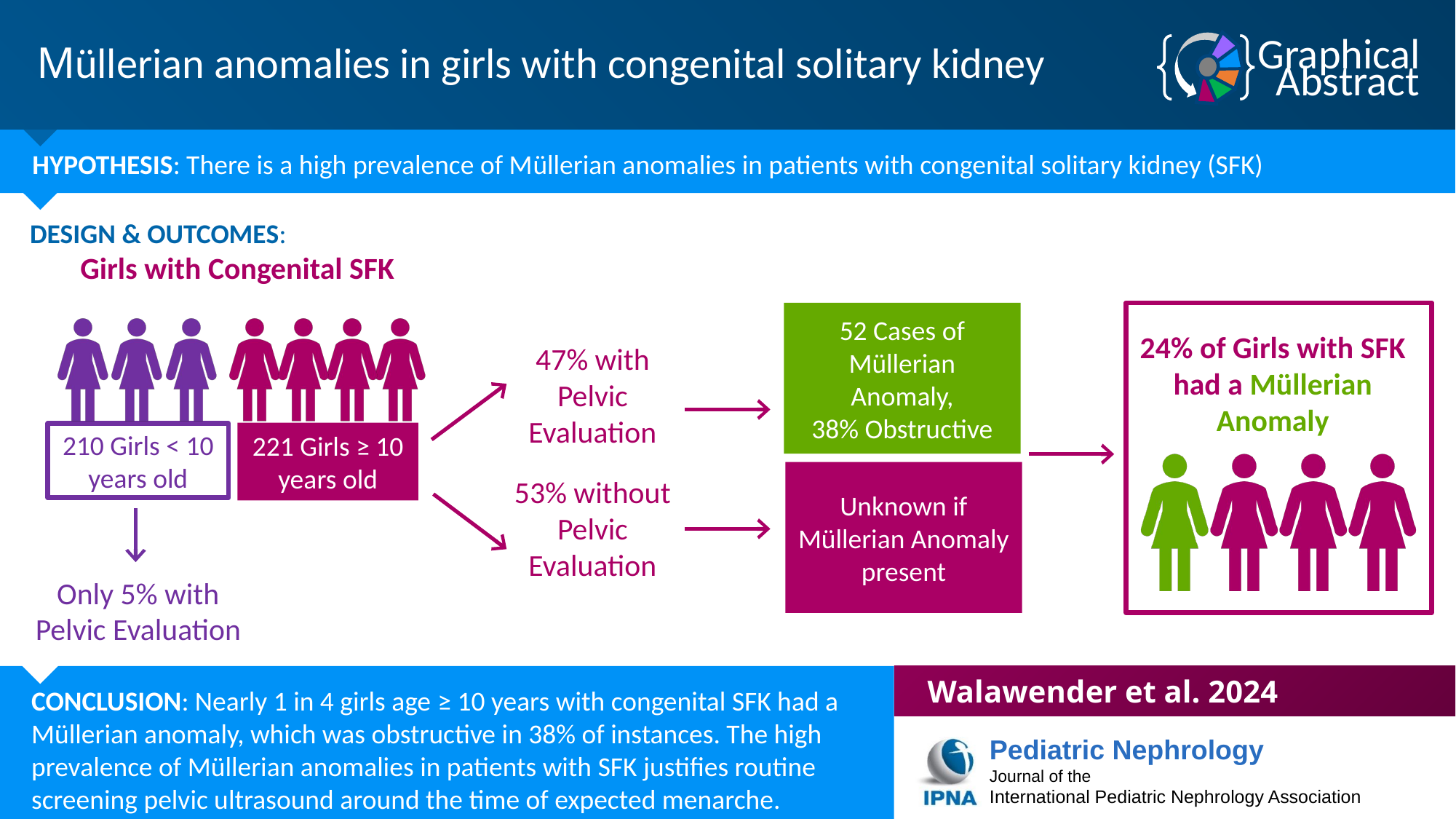

Müllerian anomalies in girls with congenital solitary kidney
HYPOTHESIS: There is a high prevalence of Müllerian anomalies in patients with congenital solitary kidney (SFK)
DESIGN & OUTCOMES:
Girls with Congenital SFK
52 Cases of Müllerian Anomaly,
38% Obstructive
24% of Girls with SFK had a Müllerian Anomaly
47% with Pelvic Evaluation
221 Girls ≥ 10 years old
210 Girls < 10 years old
Unknown if Müllerian Anomaly present
53% without Pelvic Evaluation
Only 5% with Pelvic Evaluation
Walawender et al. 2024
CONCLUSION: Nearly 1 in 4 girls age ≥ 10 years with congenital SFK had a Müllerian anomaly, which was obstructive in 38% of instances. The high prevalence of Müllerian anomalies in patients with SFK justifies routine screening pelvic ultrasound around the time of expected menarche.
